# Associative Learning Emulation in HZO-Based Ferroelectric Memristor Devices

**DOI:** 10.3390/ma18143210

**Published:** 2025-07-08

**Authors:** Euncho Seo, Maria Rasheed, Sungjun Kim

**Affiliations:** 1 Division of Electronics and Electrical Engineering, Dongguk University, Seoul 04620, Republic of Korea; 2 Department of Advanced Battery Convergence Engineering, Dongguk University, Seoul 04620, Republic of Korea

**Keywords:** neuromorphic computing, associative learning, ferroelectric memristor, short-term memory, hafnium zirconium oxide (HZO)

## Abstract

Neuromorphic computing inspired by biological synapses requires memory devices capable of mimicking short-term memory (STM) and associative learning. In this study, we investigate a 15 nm-thick Hafnium zirconium oxide (HZO)-based ferroelectric memristor device, which exhibits robust STM characteristics and successfully replicates Pavlov’s dog experiment. The optimized 15 nm HZO layer demonstrates enhanced ferroelectric properties, including a stable orthorhombic phase and a reliable short-term synaptic response. Furthermore, through a series of conditional learning experiments, the device effectively reproduces associative learning by forming and extinguishing conditioned responses, closely resembling biological neural plasticity. The number of training repetitions significantly affects the retention of learned responses, indicating a transition from STM-like behavior to longer-lasting memory effects. These findings highlight the potential of the optimized ferroelectric device in neuromorphic applications, particularly for implementing real-time learning and memory in artificial intelligence systems.

## 1. Introduction

Neuromorphic computing systems aim to replicate the information processing capabilities of the human brain by utilizing several memory devices that emulate synaptic plasticity [1,2]. Ferroelectric materials, characterized by their spontaneous polarization that can be reversed by an external electric field, have gained significant attention for their potential in non-volatile memory and neuromorphic applications [3,4,5]. These materials exhibit strong polarization retention, low-power switching, and high-speed operation, making them ideal for synaptic emulation in artificial neural networks. Among various materials showing ferroelectric properties, hafnium zirconium oxide (HZO) has emerged as a promising candidate for next-generation memory applications due to its excellent ferroelectric properties, CMOS compatibility, and tunability [6,7,8,9,10]. HZO, a fluorite-structured ferroelectric, demonstrates robust polarization switching at nanometer thicknesses, making it highly suitable for scaled memory and logic devices [11,12]. Unlike traditional perovskite-based ferroelectrics, HZO can be integrated into conventional semiconductor fabrication processes, allowing for easier adoption in modern electronic systems.

Additionally, two-terminal and three-terminal ferroelectric devices have recently gained attention in both logic and memory applications, offering advantages such as non-volatility, low power consumption, and high-speed operation [13,14,15,16,17]. These devices are actively being explored for use in novel computing architectures, further reinforcing the importance of optimizing ferroelectric materials like HZO for neuromorphic and memory-centric applications.

Recently, various types of artificial synaptic devices have been proposed for neuromorphic hardware implementations, including resistive random-access memory (RRAM), ferroelectric tunnel junctions (FTJs), and phase-change memory (PCM). RRAM devices offer advantages such as a simple two-terminal structure, fast switching speed, and multilevel storage capability. However, they often suffer from filament formation variability and poor retention characteristics, which limit their long-term reliability and learning accuracy. FTJs exhibit excellent non-volatility and fast switching behavior, but their extremely thin barrier layers and complex fabrication processes pose challenges for large-scale integration. In comparison, HZO-based ferroelectric memristors simultaneously achieve low switching voltage, low power consumption, excellent retention, and high learning accuracy, while offering superior compatibility with existing CMOS processes, making them well suited for high-density integration. Notably, HZO devices enable gradual and controllable conductance modulation through polarization switching, which is highly advantageous for precise synaptic weight tuning and achieving learning linearity. Furthermore, unlike RRAM, HZO-based memristors can emulate both long-term and short-term memory behaviors, providing a distinct advantage for implementing reliable, energy-efficient neuromorphic systems. These combined features position HZO-based ferroelectric devices as highly promising candidates for next-generation neuromorphic hardware.

Particularly, the HZO-based ferroelectric memory devices, including oxygen vacancies, have been demonstrated to exhibit superior short-term memory (STM) characteristics, making them an ideal platform for mimicking biological learning processes [18,19,20,21,22,23,24]. The ability of ferroelectric materials to maintain multiple stable resistance states, arising from polarization dependent domain configurations, enables the realization of essential synaptic functionalities, such as paired-pulse facilitation (PPF) and spike-timing-dependent plasticity (STDP), both of which are foundational to learning and adaptation in neuromorphic systems.

In particular, HfO_2_-based ferroelectric devices have demonstrated precise modulation of conductance states through controlled voltage pulses, enabling them to emulate both short-term synaptic enhancements, such as paired-pulse facilitation (PPF) and long-term spike-timing-dependent plasticity (STDP) [25,26]. For example, Yu et al. reported an Hf_0.5_Zr_0.5_O_2_-based memristor exhibiting reliable multilevel storage and distinct STDP characteristics under asymmetric pulse schemes, highlighting its viability as an artificial synapse [27]. Additionally, Boyn et al. revealed that nanoscale ferroelectric domain dynamics directly contribute to synaptic weight modulation, offering insights into the physical mechanisms underlying spike-based learning processes [28].

One of the key types of memory devices used for neuromorphic applications is the ferroelectric memristor, which differs significantly from ferroelectric tunnel junctions (FTJs) in its underlying mechanism. Unlike FTJs, which rely primarily on polarization switching across an ultrathin ferroelectric barrier, ferroelectric memristors incorporate a higher density of oxygen vacancies within the ferroelectric layer. These oxygen vacancies contribute to a more volatile retention characteristic compared to FTJs, leading to a natural decay of stored information over time [21]. While poor retention is typically considered a drawback in conventional memory applications, it is actually advantageous for neuromorphic computing, where short-term plasticity and associative learning require transient memory effects [29].

The limited retention in ferroelectric memristors makes them particularly well suited for mimicking biological synaptic functions, where memory traces gradually fade unless reinforced by repeated stimuli. This characteristic is crucial for implementing classical conditioning-based learning, such as Pavlov’s dog experiment, where associations between stimuli must be reinforced over multiple training cycles. Compared to FTJs, ferroelectric memristors offer more dynamic memory behavior, better aligning with the time-dependent plasticity mechanisms observed in biological neurons. The ability to encode and gradually forget information without requiring external resets makes ferroelectric memristors a promising candidate for real-time, energy efficient neuromorphic systems. Furthermore, associative learning mechanisms enabled by such memory devices can be directly applied to neuromorphic applications, including spiking neural networks and real-time adaptive learning systems [29]. In this study, we focus on the STM properties of the HZO device and its ability to reproduce associative learning behavior, specifically Pavlov’s dog experiment. The experiment is a classic example of conditioned learning, where a neutral stimulus becomes associated with an unconditioned response through repeated training. Here, we leverage the short-term synaptic plasticity of our ferroelectric device to mimic this process, demonstrating its potential for neuromorphic applications.

## 2. Experiments

The Mo/HZO/n^+^ Si metal–ferroelectric–semiconductor (MFS) structure was fabricated using atomic layer deposition (ALD) to achieve a 15 nm-thick HZO layer as shown in Table 1. The composition ratio of HfO_2_ to ZrO_2_ was maintained at 1:1 to optimize ferroelectric performance. The device underwent rapid thermal annealing (RTA) to promote orthorhombic phase formation. The electrical characteristics were measured using a semiconductor parameter analyzer, where the polarization–voltage (P-V) response was obtained using the positive-up-negative-down (PUND) method to confirm the presence of a stable ferroelectric response. The electrical characteristics of devices were assessed using the Keithley 4200-SCS semiconductor parameter analyzer and the 4225-PMU pulse measurement unit from Keithley Instruments in Cleveland, OH, USA. The bias was applied to the top electrode (Mo), while the bottom electrode (Si) remained grounded.

## 3. Results and Discussions

**Figure 1:**
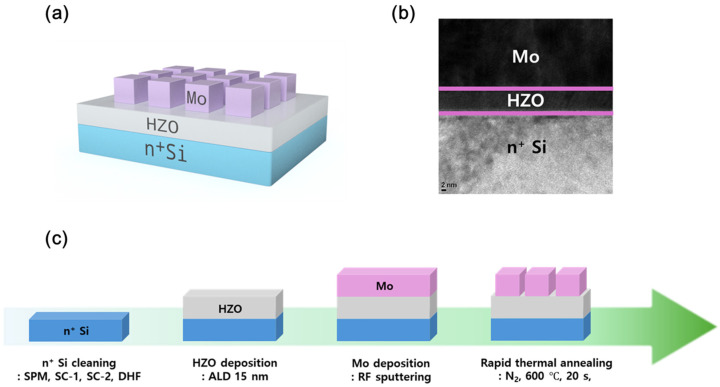
(**a**) Schematic illustration of the MFS device structure composed of Mo/HZO/n^+^ Si stack. (**b**) Cross-sectional transmission electron microscopy (TEM) image showing the layered configuration and thickness of the HZO film. (**c**) Fabrication process flow of the ferroelectric memristor using atomic layer deposition (ALD) and rapid thermal annealing (RTA).

Figure 1a illustrates the device configuration utilized in this study, where the top electrode is composed of Mo, the ferroelectric layer consists of HZO, and the bottom electrode is made of n^+^ Si. Figure 1b presents a transmission electron microscopy (TEM) image that captures the cross-sectional view of the fabricated device, highlighting the atomic layer deposition (ALD) of the thin HZO film.

Figure 1c illustrates the fabrication process of the device. Initially, the bottom electrode consisted of highly doped n^+^ Si. To eliminate organic contaminants from the substrate surface, a sulfuric acid peroxide mixture (H_2_SO_4_:H_2_O_2_ = 4:1) was used. Natural oxides were then removed using diluted hydrofluoric acid (DHF) in a 1:100 HF:H_2_O ratio, ensuring thorough cleaning of the n^+^ Si substrate. Next, HZO films with thicknesses of 10, 15, and 20 nm were deposited via atomic layer deposition (ALD). The deposition was performed at a stage temperature of 290 °C, maintaining a 1:1 ratio of HfO_2_ to ZrO_2_. The HfO_2_ and ZrO_2_ precursors used were tetrakis(ethylmethylamino)hafnium (TEMAHF) and tetrakis(ethylmethylamino)zirconium (TEMAZ), respectively, with ozone (O_3_) serving as the reaction gas. Following the HZO deposition, the top Mo electrode was deposited via radiofrequency sputtering to a thickness of 100 nm. Finally, rapid thermal annealing (RTA) was performed at 600 °C in an N_2_ atmosphere for 20 s.

**Figure 2:**
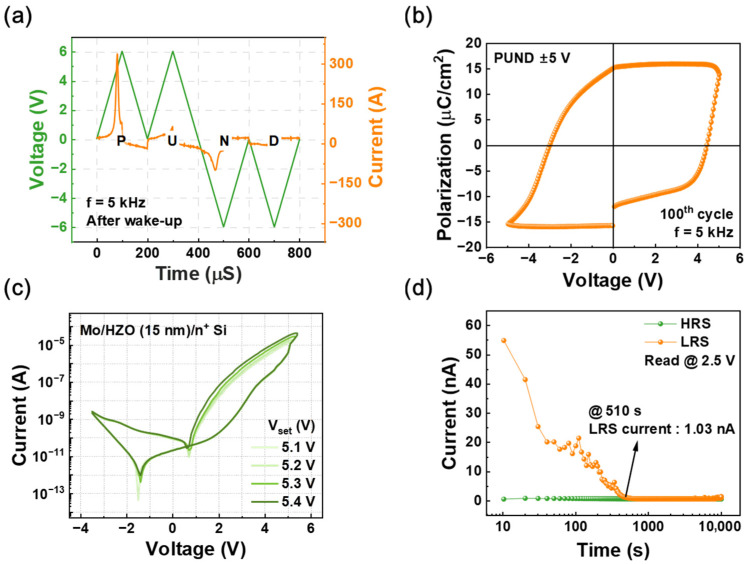
(**a**) Polarization–voltage (P–V) curve measured using the PUND method, confirming the ferroelectric behavior of the HZO layer. (**b**) Converted polarization data using the same PUND pulse waveform. (**c**) Current–voltage (I–V) characteristics exhibiting hysteresis due to oxygen vacancy migration in devices with different HZO thicknesses. (**d**) Retention characteristics showing exponential decay of current over 700 s, indicating short-term memory behavior influenced by defect-assisted conduction.

Figure 2a presents the polarization–voltage (P–V) curve measured using the positive-up-negative-down (PUND) method. The corresponding PUND waveform is also depicted in Figure 2a. P–V curves are shown in Figure 2b, converted from the PUND method. When a pulse amplitude of 6 V was applied at a frequency of 5 kHz, the average remnant polarization (2P_r_) was measured to be about 43 μC/cm^2^. The PUND method is a widely used technique for accurately measuring the remnant polarization in ferroelectric materials by eliminating non-ferroelectric contributions, such as leakage currents and charge injection effects. It applies a sequence of voltage pulses to separately measure the switchable and non-switchable polarization components, enabling the precise evaluation of the intrinsic ferroelectric properties.

Figure 2c illustrates the current–voltage (I–V) characteristics of devices with varying thicknesses, measured using a dual direct current sweep. The voltage initially increased from −3.5 V to 5.2 V in 0.1 V increments and subsequently decreased from 5.2 V to −3.5 V in 0.1 V increments. Notably, the I–V curves exhibit a pronounced hysteresis effect, which can be attributed to the migration of oxygen vacancies within the HZO layer, a common characteristic in memristive devices. Unlike ideal ferroelectric switching, where the hysteresis primarily arises from domain nucleation and growth, the observed I–V response suggests a mixed mechanism involving both ferroelectric switching and resistive switching effects. The presence of oxygen vacancies could play a crucial role in modulating the device resistance, leading to charge trapping and de-trapping effects that contribute to hysteresis. These vacancies can drift under an applied electric field, altering the local built-in field and effectively influencing charge transport characteristics.

Furthermore, while the PUND method is typically employed to isolate pure ferroelectric polarization by removing non-switching components, such as leakage and conduction currents, as shown in Figure 2a, our results indicate that the measured polarization response is not solely due to intrinsic ferroelectric switching. Instead, the involvement of defect-mediated conduction, particularly through the redistribution of oxygen vacancies, suggests that part of the remnant polarization signal may be influenced by resistive switching rather than purely ferroelectric behavior. This interplay between ferroelectric and memristive effects highlights the necessity of a more comprehensive analysis to accurately interpret the switching dynamics of the device.

Additionally, the leakage current plays a significant role in the transport characteristics, leading to short-term memory (STM)-like behavior in the device. The continuous charge redistribution caused by leakage pathways, particularly through oxygen vacancy-assisted conduction, results in a gradual modulation of the current, resembling analog synaptic weight updates in neuromorphic systems. This leakage-driven STM effect suggests that the device does not exhibit an abrupt bistable switching mechanism typical of pure ferroelectric capacitors but rather a gradual conductance change reminiscent of resistive switching or electrostatic charge trapping effects. Figure 2d shows the normalized current as a function of time. The current exhibits an exponential decay over a duration of 700 s. The current exhibits a decreasing trend for all thicknesses, indicating relaxation behavior. This result suggests stronger charge trapping or leakage effects. This trend highlights the influence of the deposited film on retention characteristics, likely due to variations in defect density, polarization stability, or leakage pathways within the HZO layer over the measured time period.

**Figure 3:**
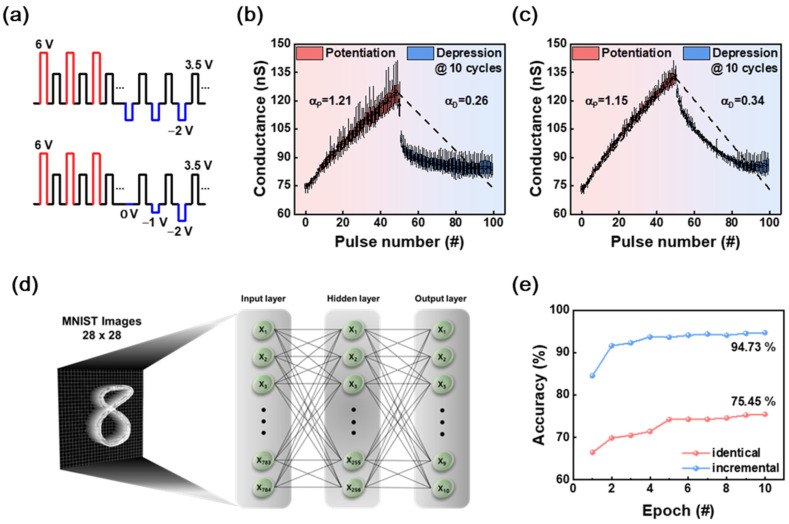
(**a**) Applied pulse schemes for potentiation and depression under identical and incremental conditions. (**b**) Synaptic conductance evolution for identical pulse application showing a steeper depression slope (α_d_ = 0.26). (**c**) Conductance modulation with incremental depression pulses, resulting in improved linearity (α_d_ = 0.34). (**d**) Schematic of the neural network architecture used for MNIST classification, consisting of input, hidden, and output layers. (**e**) Classification accuracy comparison between identical and incremental pulse schemes over 10 epochs, where the incremental scheme achieved 94.73% accuracy due to better linearity of weight updates.

Figure 3a–c illustrates the potentiation and depression (P/D) characteristics, which are fundamental mechanisms in learning and memory functions of the human brain. P/D describes two states of synaptic modulation, as follows: potentiation, where synaptic strength increases, and depression, where synaptic strength decreases in response to repeated stimuli. Figure 3a shows the waveforms used for both identical and incremental P/D experiments. For the identical pulse scheme, 50 potentiation pulses at 6 V are followed by 50 depression pulses at a fixed −2 V. In contrast, the incremental pulse scheme applies 50 potentiation pulses at 6 V followed by 50 depression pulses that progressively increase in magnitude from 0 V to −2 V. The pulse width and interval are kept constant throughout both experiments.

Figure 3b,c displays the corresponding conductance responses to these pulse schemes. In both cases, the device exhibits an increase in conductance during potentiation followed by a decrease during depression. The slope parameters αₚ and α_d extracted from the linear fitting lines reflect the modulation efficiency of synaptic weights. Notably, the incremental depression scheme slightly improves linearity during weight updates; although, both approaches exhibit sharp conductance drops during the depression phase, suggesting a need for further optimization.

Figure 3d presents the schematic of a fully connected neural network architecture designed to evaluate the applicability of the proposed device for neuromorphic computing. The network consisted of 784 input neurons corresponding to 28 × 28-pixel MNIST handwritten digit images, 256 hidden neurons, and 10 output neurons representing digit classes. The synaptic weights between neurons were implemented using the conductance values extracted from the experimental potentiation/depression (P/D) behavior. The ReLU activation function was applied to the hidden layer, while the SoftMax function was used in the output layer to obtain class probabilities. The network was trained using the cross-entropy loss function and the backpropagation algorithm. Based on the device’s LTP/LTD characteristics, synaptic weights were updated through both identical pulse schemes and incrementally increasing amplitude pulse schemes. The classification accuracy was evaluated over a total of 10 training epochs.

Figure 3e compares the classification accuracy of networks trained using the identical and incremental pulse schemes over 10 training epochs. The incremental pulse scheme achieves a final accuracy of 94.73%, while the identical scheme reaches 75.45%. This significant improvement is attributed to the enhanced linearity and stability of synaptic updates in the incremental scheme. By tailoring the potentiation and depression pulse schemes, we not only improved the conductance update linearity but also identified an optimal dynamic range that minimizes read/write noise. This effectively defines a reliable and valid memory state window suitable for neuromorphic applications under non-ideal device conditions. These findings emphasize the critical role of pulse scheme engineering in optimizing memristor-based synaptic devices for efficient and accurate neuromorphic learning applications.

**Figure 4:**
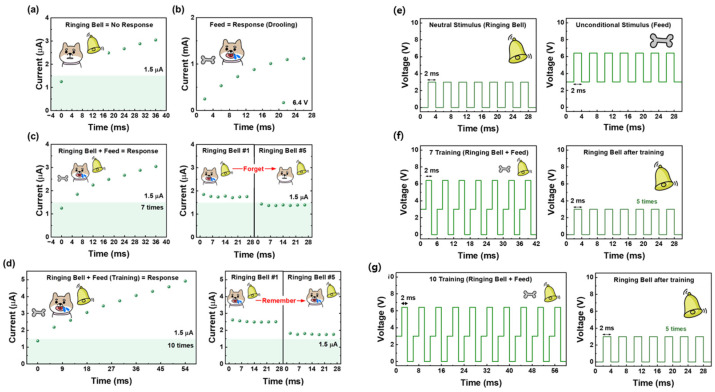
(**a**) Pre-conditioning stage where the device shows no response to a neutral stimulus (bell alone). (**b**) Application of the unconditioned stimulus (food) results in a significant increase in current above the threshold, representing an innate biological response. (**c**) After 7 training cycles with paired stimuli, the device initially exhibits a conditioned response to the bell alone, which fades over time, reflecting memory extinction. (**d**) With 10 training repetitions, the device maintains the conditioned response over repeated tests, indicating improved memory retention through reinforced learning. (**e**) Pulse waveform used for presenting only the bell stimulus and pulse sequence for applying only the food stimulus. (**f**) Training protocol consisting of 7 repetitions of paired bell and food pulses. (**g**) Extended training sequence of 10 repetitions followed by bell-only pulses to evaluate memory recall, effectively emulating the temporal structure of associative learning found in biological systems.

Figure 4 demonstrate the emulation of Pavlov’s classical conditioning experiment for associative learning using a metal–ferroelectric–semiconductor (MFS) device. This experiment is a representative example of how artificial synapses can replicate biological learning behaviors through repeated exposure to paired stimuli. Specifically, the conditioning process mimics how a biological system forms associations between a neutral and an unconditioned stimulus through repeated co-occurrence, ultimately leading to a conditioned response. The pulse schemes applied to the device during each stage of the experiment are detailed in Figure 4a–d, highlighting how electrical stimulation can simulate cognitive processes [30].

In the pre-conditioning phase shown in Figure 4a, a neutral stimulus—represented by a ringing bell—is applied to the device. This stimulus alone does not produce any measurable response, simulating the natural behavior in biological systems where an unfamiliar stimulus initially lacks meaning. This reflects the baseline state of the synaptic device prior to any learning, where the current remains below the recognition threshold. The threshold for a learned response in this experiment was defined as 1.5 μA.

Figure 4b displays the unconditioned response elicited by the application of an unconditioned stimulus, such as food. Upon receiving this input, the device shows a significant increase in current, indicating a strong intrinsic response without prior training. This stage serves to validate that the device can differentiate between meaningful and non-meaningful stimuli, and that the current level can reliably reflect synaptic activation.

Figure 4c,d illustrates the progression of classical conditioning through repeated co-application of the neutral and unconditioned stimuli. In Figure 4c, after seven training repetitions where the bell and food stimuli were paired, the neutral stimulus (bell) alone is applied. Initially, the device responds with a current above the threshold, indicating successful associative learning. However, as additional bell-only pulses are applied without reinforcement, the response gradually diminishes, and the current falls below the threshold by the fifth application. This behavior replicates the biological process of memory extinction, where learned associations fade in the absence of reinforcement, and demonstrates the device’s ability to exhibit time-dependent memory decay.

In contrast, Figure 4d shows the outcome when the number of training sessions is increased to ten. In this case, the device exhibits a stable and sustained response to the bell stimulus alone, even after the fifth application. The current remains consistently above the defined threshold, indicating stronger and more persistent memory formation. This result confirms that the number of training repetitions plays a critical role in modulating memory retention in the device, analogous to long-term potentiation in biological neurons.

Figure 4e–g provides a detailed overview of the electrical pulse schemes applied during each stage of the experiment—pre-conditioning, training, and post-conditioning. The sequences visually represent the pairing of stimuli and the timing of their delivery. The bell and food pulses are temporally aligned during training and separated during the testing phase to evaluate memory recall. These pulse patterns are designed to mimic temporal association mechanisms in real synaptic networks [31].

Together, these results confirm that the MFS device not only demonstrates short-term memory behavior but also successfully mimics the essential dynamics of associative learning. The ability to encode, recall, and forget information through pulse-driven synaptic modulation positions ferroelectric memristor devices as strong candidates for real-time neuromorphic computing platforms that require biological fidelity in learning processes.

## 4. Conclusions

In this study, we demonstrated that a 15 nm HZO-based ferroelectric device possesses outstanding short-term memory (STM) behavior and can reproduce Pavlovian associative learning. The device effectively mimics the formation, retention, and gradual extinction of conditioned responses, closely resembling the functional dynamics of biological synapses. These capabilities suggest strong potential for application in neuromorphic computing systems. Our findings emphasize the critical role of STM mechanisms in enabling associative learning within MFS-based architecture. Furthermore, the results show that STM-driven neuromorphic devices can emulate key cognitive functions observed in nature, including learning processes, memory consolidation, and forgetting. Leveraging these volatile memory characteristics, memristor technologies hold great promise for constructing biologically inspired learning systems, with significant implications for next-generation artificial intelligence and adaptive computing platforms. In the future, integrating long-term memory functionalities alongside the short-term memory characteristics demonstrated in this study will be essential for the development of more complete neuromorphic systems. Additionally, we plan to incorporate unsupervised learning schemes and three-dimensional stacked ferroelectric memristor array architectures to enhance memory density and parallel processing efficiency, ultimately advancing toward brain-inspired artificial intelligence computing platforms. In addition, to improve the controllability of transient leakage currents in STM operations, future work will explore device engineering approaches, such as far-gate structures.

## Figures and Tables

**Table 1:** Key parameters of the fabrication process.

Key Parameters	Fabrication Details
HZO thickness	15 nm deposition with ALD
Composition ratio (HfO_2_:ZrO_2_)	1:1 (precursors: TEMAHF for HfO_2_, TEMAZ for ZrO_2_)
Annealing conditions	600 °C, 20 s, N_2_ atmosphere (RTA)
average remnant polarization (2P_r_)	43 μC/cm^2^ (measured by PUND at 6 V, 5 kHz)

## Data Availability

The original contributions presented in this study are included in the article. Further inquiries can be directed to the corresponding author.
